# Microbial degradation of aristolochic acid I by endophytic fungus A.h-Fs-1 of *Asarum heterotropoides*

**DOI:** 10.3389/fmicb.2022.917117

**Published:** 2022-07-22

**Authors:** Xiaohan Wang, Dan Jiang, Qijin Shi, Guangxi Ren, Chunsheng Liu

**Affiliations:** School of Chinese Materia Medica, Beijing University of Chinese Medicine, Beijing, China

**Keywords:** aristolochic acid I, *Asarum heterotropoides*, endophytic fungi, *Neocosmospora solani*, *o*-demethylation

## Abstract

Asari Radix et Rhizoma is commonly used in classic prescriptions of herbal medicine in several Asian countries for resuscitation, pain relief, and sore treatment, and *Asarum heterotropoides* (*A. heterotropoides*) is an important source material of Asari Radix et Rhizoma. However, the plants of the Asari Radix et Rhizoma and some plants in *Asarum* spp. contain aristolochic acid I (AAI), which is considered as a carcinogen. The objective of the current study is to detoxify *Asarum* spp. through microbial degradation of AAI in order to ensure drug safety. Based on the observation of the close correlation between endophytic fungi of *A. heterotropoides* and AAI, we identified an AAI-degrading fungus and screened for candidate genes involved in AAI degradation. Full-length *O*-demethylase genes (*ODMs*) were cloned including *A.h-ODM-5*, *Fs-ODM-4,* and *Fs-ODM-1*, and their ability to degrade AAI was tested *in vitro*. The results showed that the AAI-degrading fungus was identified as *Neocosmospora solani* (A.h-Fs-1, endophytic fungi of *A. heterotropoides*), and verified the capability of specific *O*-demethylation to modify the structure of AAI. We further identified the functional ODMs in A.h-Fs-1 capable of degrading AAI and uncovered the AAI degradation mechanism of A.h-Fs-1. The microbial degradation of AAI demonstrated in the present study offers a new method to detoxify plant materials used for herbal medicine, and would enhance the regulation of toxic ingredients content in herbal medicine source materials.

## Introduction

The medicinal Asari Radix et Rhizoma source plants specified in the Chinese Pharmacopoeia include the dry roots and rhizomes of *Asarum heterotropoides* Fr. Schmidt var. *mandshuricum* (Maxim.) Kitag., *A. sieboldii* Miq. var. *seoulense* Nakai., and *A. sieboldii* Miq (Chinese Pharmacopoeia Commission, 2020), which are commonly used for resuscitation, pain relief, and sore treatment (Luo, 2017). The genus *Asarum* L. is typically distributed in temperate regions in the Northern Hemisphere, with the center of diversity in Eastern Asia (Lim et al., 2017). Since ancient times, *Asarum* spp. has been widely used as herbal medicine to treat aphthous stomatitis, toothache, and gingivitis (Yamaki et al., 1996) in traditional medical practices in China, Korea, and Japan. However, *Asarum* spp. source plants contain nitrophenanthrene organic acids called aristolochic acids (AAs) that were verified to be toxic. The toxicity of AAs was first reported in 1993 by Vanherweghem et al. (1993), who found that Belgian women suffered renal interstitial fibrosis and renal failure after taking weight-loss drugs containing AAs. The discovery triggered worldwide research on the toxicity mechanisms of AAs and safe and rational use of drugs containing AAs. In 2017, Ng et al. (2017) suggested that AAs are related to liver cancer in Asians and questioned the safety of traditional Chinese medicines containing AAs. The toxicity of AAs leads to the exclusion of *Aristolochiaceae juss.* Medicinal materials from the 2020 edition of the Chinese Pharmacopoeia, except for Asari Radix et Rhizoma which has low AAs content in its roots and rhizomes and has no alternatives yet to achieve the same medicinal effect (Chinese Pharmacopoeia Commission, 2020; Supplementary Figure S1).

AAs exhibit nephrotoxicity, liver toxicity, carcinogenesis, and mutagenesis and are classified as grade I carcinogens (Wang et al., 2018). Further studies indicate that AAs have multiple types of molecular structure, with aristolochic acid I (AAI) being the most toxic (Balachandran et al., 2005; Supplementary Figure S2). The toxicity of AAI is closely related to the substituent nitro, methoxy, and hydroxyl groups in its molecular structure. AAI is rapidly absorbed after entering the human body and shows an organ-specific distribution that is mainly concentrated in liver and kidney tissues (Gao et al., 2017). The content of AAI in *Asarum* spp. source plants, which are commonly used in herbal medicine, can reach an alarming level as high as 3,243 μg·g^−1^, as reported by Gao et al. (2006). Therefore, to guarantee medicinal and clinical safety, it is imperative to remove the content of AAI in *Asarum* spp. used as raw material for the pharmaceutical industry.

Detoxification of *Asarum* spp. by removing the AAI compound remains challenging and is still to be developed yet. Microorganisms may shed some light on this challenge because they are capable of decomposing and transforming a wide variety of substances, including some herbal endophytes (Ai et al., 2019). Microorganisms have been used to ferment traditional Chinese medicines to make their active substances more readily available or metabolizable to obtain more active substances (Wang et al., 2010; Wu, 2015; Chen, 2016). Cao et al. (2015) used 10 types of microorganisms to biotransform and detoxify 12 medicinal materials *via* liquid fermentation technology. Studies (Yang, 2009; Pan et al., 2013; Bao et al., 2017) have shown that the endophytic fungus *Aspergillus* C1-Y7-4 and its spore suspension from *Stellera Chamaejasme* L naturally degrade flavonoids and coumarins in the original plant. Zikmundov et al. (2002) isolated four strains of endophytic fungi from *Acanthaceae* spp. and analyzed the ability of *Fusarium sambucinum* to degrade 2-hydroxy-1, 4-benzoxazin-3-one into small molecular compounds. Interestingly, Liu et al. (2010) added 20 fungal species to *Radix aristolochiae* (dry root of *Aristolochia debilis*) as a substrate for solid-state fermentation, and 13 fungal species were able to degrade AAI to various extent. These studies collectively provide new perspectives to degrade AAI compound in *Asarum* spp. through microorganism decomposition and transformation.

In the present study, we aim to detoxify *Asarum* spp. by identifying endophytic fungus that is able to decompose its toxic compound AAI, and elucidate the mechanism of AAI degradation by endophytes, which has not been reported yet. More specifically, we applied and validated the approach of demethylation, through which AAI is naturally decomposed in human body, to allow the endophytic fungus to effectively degrade AAI compound. Our study not only advances the understanding of the molecular mechanism of endophyte detoxification, but also provides a new method to detoxify herbal medicine for improved drug safety.

## Materials and methods

### Plant materials

**Figure 1:**
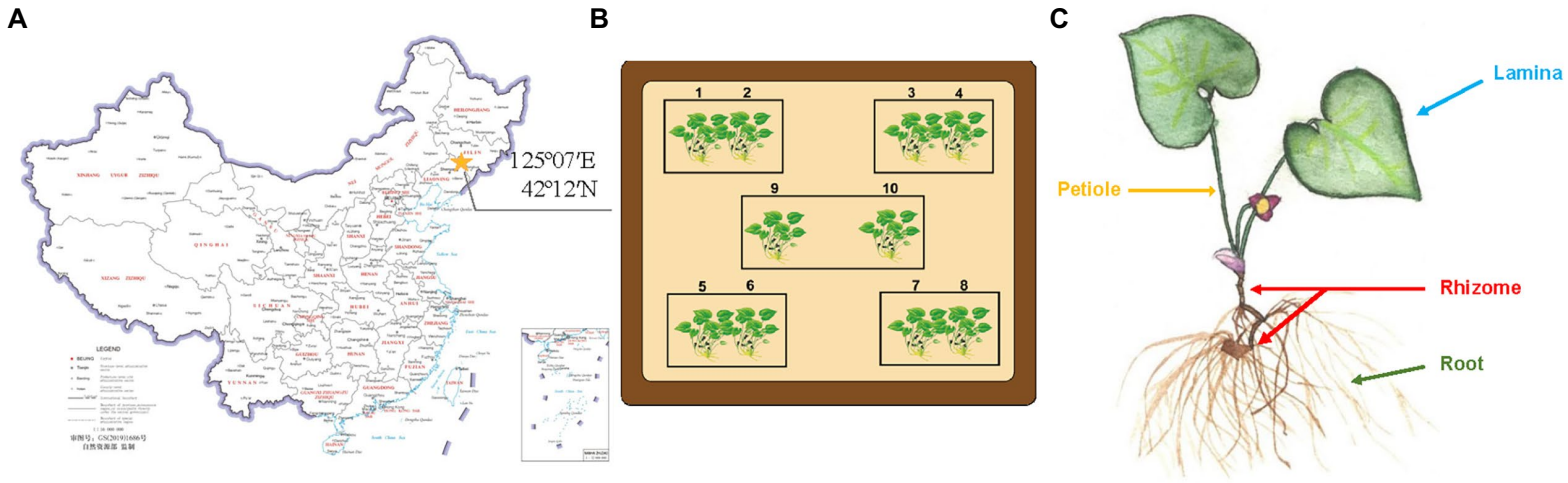
Sample collection of *Asarum heterotropoides*. **(A)** Location of *A. heterotropoides* sampling points on map. **(B)** Distribution of all the sampling points. **(C)** Different niches from which *A. heterotropoides* was sampled.

Plant materials [*A. heterotropoides* Fr. Schmidt var. *mandshuricum* (Maxim.) Kitag.] were sampled on April 28, 2019 from Gaojiagou (42°12′N, 125°07′E), Qingyuan Manchu Autonomous County, Fushun City, Liaoning province, Northern China, as shown in the map in Figure 1A. Five plants were collected from each of the 10 sampling points (Figure 1B), and each sample was composed of the same niche (root, rhizome, leaf, and petiole; Figure 1C) from the five plants of the same sampling point, making a total of 40 samples. Three-quarters of each sample material were directly used for the screening of endophytes in this study and the remaining one-quarter of each sample was transported to a company (Novogene, Beijing) for high-throughput sequencing and analysis (Supplementary Material 1).

### Screening endophytic fungi for degrading AAI in *Asarum heterotropoides*

The 40 fresh plant samples of *A. heterotropoides* were rinsed and divided into groups of roots, rhizomes, laminae, and petioles (Figure 1C). After that, the samples were first treated with 0.1% mercury chloride for 12 min (the laminae were treated for 8 min) and then with 75% ethanol for 1 min. The samples were further rinsed with sterile water and then dried using sterile filter paper (Shu et al., 2021). Under aseptic conditions, the laminae were cut into 1 × 1 cm^2^ pieces, and the roots, rhizomes, and petioles were cut into 1 cm lengths. The sample surface was verified to be completely disinfected using tissue blotting and rinse solution detection methods (Schulz et al., 1993). After cutting, the samples were placed on a phosphate-buffered saline (PBS, pH 5.8) solid medium plate and cultured at a constant temperature of 28°C for 5 days in the dark (Bhojwani and Razdan, 1983; Liu and Ji, 2009). After 5 days of culture, the plant tissues were placed in 50 ml of an AAI basic inorganic salt liquid medium (AAI standard solution: 0.515 mg/ml AAI) and cultured at 28°C in the dark (roots and rhizomes were peeled to avoid contamination; Xiang, 2019). The obtained endophytic fungi were transferred to AAI-coated inorganic salt solid medium plates and incubated for at least 7 days. Next, according to the color and morphology of the colonies, we selected hyphae that grow vigorously on the surface for further experiments. A single colony was selected, cultured, and purified repeatedly (Most of the mycelium will grow at the bottom of the medium, and a small part of the mycelium will grow on the surface of the medium. We selected the mycelium with vigorous surface growth for purification).

### Identification of the screened endophytic fungus

Fungal hyphae were placed on a slide, stained with a lactic acid phenol cotton blue staining solution, and covered with a cover glass for observation under a microscope (using cedar oil for immersion under the oil lens, BX53; Olympus, Tokyo, Japan). Amplification of the *tef-1α* gene and ITS regions was conducted using the primer pair *ef1* and *ef2* for *tef-1α* (O’Donnell et al., 1998) and ITS1 and ITS4 for the ITS regions (White et al., 1990; Supplementary Table S1). The target genes were amplified using PCR (see Supplementary Table S2 for PCR amplification system), after which the amplicons were detected and recovered using 1% agarose gel electrophoresis. PCR products were sent for sequencing to a service provider. The aligned sequences were blasted in two genome databases, GenBank and *Fusarium*-ID, to identify the strain. In this study, a phylogenetic tree was generated using maximum parsimony in MEGA7.0 (Kumar et al., 2016). Bootstrap values for the maximum parsimony tree (MPT) were calculated for 2,000 replicates. The edited ITS and *tef-1α* sequences were compared with other available *Fusarium* species sequences in GenBank. Furthermore, the sequences of some known species were downloaded from GenBank and *Fusarium*-ID, and used to reconstruct a combined ITS region and *tef-1α* phylogenetic trees. For phylogenetic analysis, 12 (ITS) and 4 (*tef-1α*) taxa were included in the combined dataset, and *Glacial ice basidiomycete* (ITS, AF261656.1) and *Fusarium mori* (*tef-1α*, NRRL 52773) were used as an outgroup.

The fungal spores were collected by scraping the hyphae with sterile water and counted using a hemocytometer under a microscope. The spore suspension was diluted to a concentration of 10^8^ cells/mL, and then 4.5 ml of the diluted suspension was added to 25.5 ml of (15% V/V inoculum) liquid fermentation medium [basic inorganic salt liquid medium: 300 μl AAI standard solution (0.515 mg/ml) per 50 ml of medium; Pan et al., 2013]. The solution was then cultured at 28°C (200 rpm) for 5 and 14 days in a shaker to obtain A.h-Fs-1 fermentation broth, which was centrifuged at 10,625 × *g* for 10 min. After centrifugation, 10 ml of the supernatant was collected and freeze-dried. Next, the pellets were reconstituted in 1 ml methanol, filtered through a 0.22-μm microporous membrane, and transferred to a sample vial for later use (Wei, 1979; Consilio Florarum Cryptogamarum Sinicarum Academiae Sinicae Edita, 2004).

### Cloning of *FS-ODM-1*, *FS-ODM-4*, and *A.h-ODM-5*

**Figure 2:**
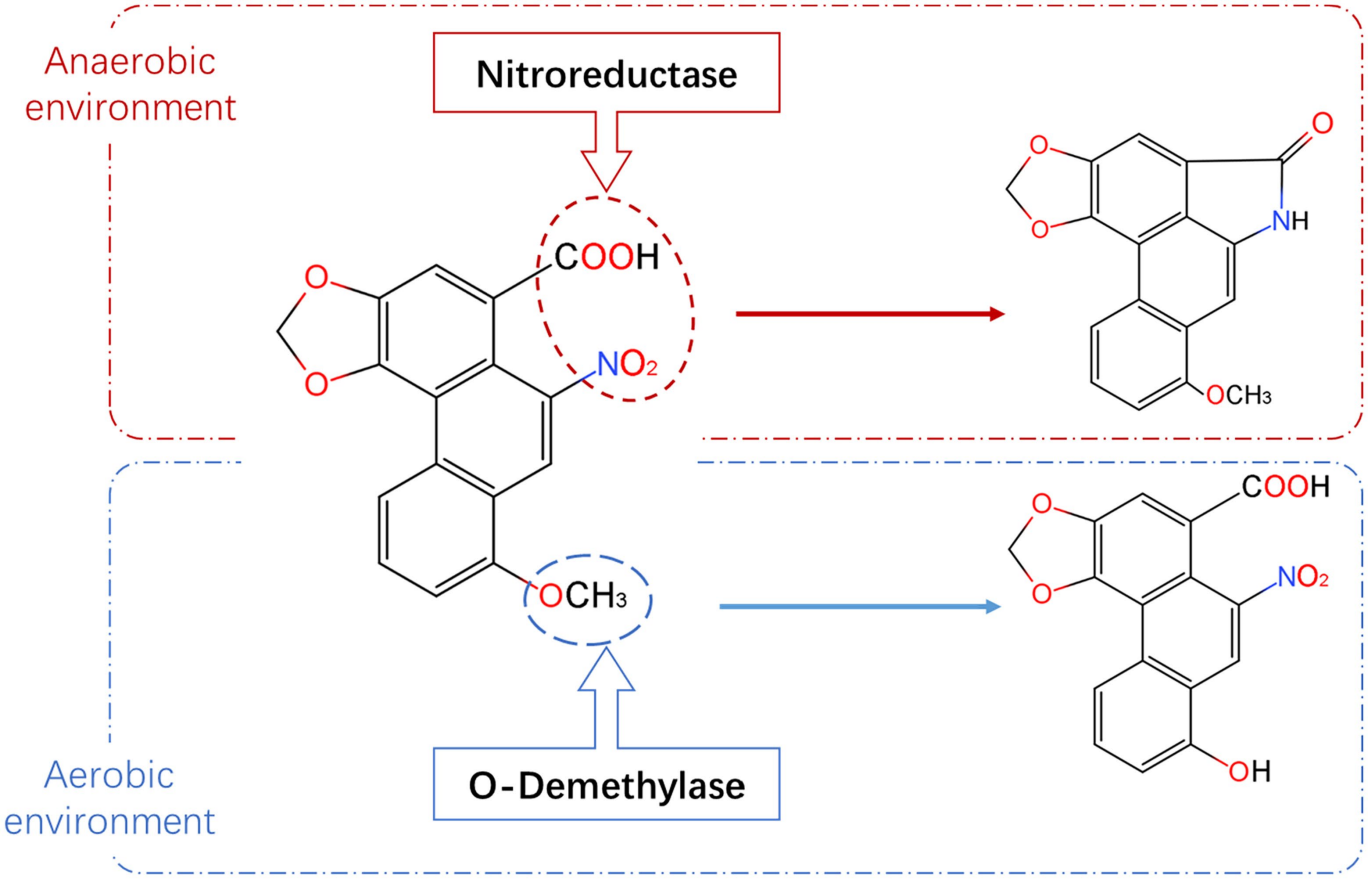
Degradation pathway and metabolites of AAI. The red dotted box represents the metabolic pathway and metabolites of AAI under anaerobic condition, and the blue box represents the metabolic pathway and metabolites of AAI under aerobic condition (information summarized and adapted from Pfau et al., 1990; Shibutani et al., 2010; Levová et al., 2011; Stiborová et al., 2015 Chang et al., 2017; Anger et al., 2020).

Based on the molecular structure (Figure 2; Pfau et al., 1990; Shibutani et al., 2010; Levová et al., 2011; Stiborová et al., 2015; Chang et al., 2017; Anger et al., 2020) and carcinogenic properties of AAI, candidate genes that can degrade AAI were screened. The *N. solani* genome database (PRJNA368786) and *A. heterotropoides* transcriptome database (PRJNA477885) on NCBI were used to design gene-specific primers (Supplementary Table S1) using Primer Premier 5.0. Primers were subsequently synthesized by Sangon Biotech (Shanghai, China). RNA was extracted from *A. heterotropoides* and A.h-Fs-1 with TRIZOL (Takara Bio, Beijing, China) and used for complementary DNA (cDNA) synthesis. The target genes were amplified using PCR (see Supplementary Table S2 for PCR amplification system), after which the amplicons were detected and recovered using 1% agarose gel electrophoresis. Target bands were recovered using an OMEGA Gel Extraction Kit (Omega Bio-tek, Norcross, GA, United States), and the purified PCR products were cloned into the pEASY^®^-Blunt cloning vector (TransGen Biotech, Beijing, China). Ligation reactions were transformed into *Escherichia coli* Trans1-T1 competent cells, spread onto LB plates supplemented with ampicillin, and incubated overnight at 37°C. Colony PCR was conducted to identify positive colonies for inoculation and subsequent plasmid isolation. Finally, the isolated plasmids were sequenced by Sangon Biotech (Shanghai, China).

### Bioinformatic analysis of *FS*-ODM-1, *FS*-ODM-4, and *A.h*-ODM-5

The similarity of nucleotide and amino acid sequences was analyzed using NCBI^1^ BLAST tool. More specifically, we used a series of publicly available toolkits to perform bioinformatic analysis, including ORF Finder^2^ to find open reading frames, ExPASy^3^ to predict the physical and chemical properties of the translated proteins, TMHMM Server v.2.0^4^ for protein transmembrane domain analysis, SinalP-5.0 Server^5^ for protein sequence signal peptide prediction analysis, NCBI-CDD^6^ for protein domain analysis, SOPMA^7^ for protein secondary structure analysis, I-TASSER and Swiss Model^8^^,^^9^for 3D structural modeling, and PyMOL software to predict the tertiary structure of the protein. MEME^10^ and TBtools (Chen et al., 2020) were used to analyze the conserved motifs of ODMs, and DNAMAN 9 software was used for comparison with the amino acid sequences of other plants. The maximum likelihood phylogenetic tree was constructed using MEGA 7.0 software with 1,000 bootstrap repetitions.

### Heterologous expression of *FS*-ODM-1, *FS*-ODM-4, and *A.h*-ODM-5 and AAI degradation assays

The sequenced pEASY^®^-Blunt E1 expression vector (Ampicillin resistance, 100 mg/ml, addition amount is 0.1%V/V) harboring *FS-ODM-1*, *FS-ODM-4*, and *A.h-ODM-5* was transformed into *E. coli TransB* (DE3) cells for heterologous protein expression. When the OD_600_ of the bacterial cultures reached approximately 0.6, isopropyl-beta-D-thiogalactopyranoside was added to form a solution at medium concentration of 0.1 mmol/l. The solution was divided into two portions and they were induced for protein expression under different conditions, with one portion at 16°C for 16 h and the other at 37°C for 4 h (Gao and Sun, 2021). The controls were formed by uninduced and blank strains (NC, negative control, pEASY®-Blunt E1 expression vector), and protein expression was detected using sodium dodecyl sulfate-polyacrylamide gel electrophoresis (SDS-PAGE) and Western Blot (WB).

Following culture at 16°C for 16 h, a large amount of fermentation broth was obtained. The bacterial solution was centrifuged at 9380 *× g* for 10 min at 4°C, after which the pellet was washed twice with 1.5 ml of Phosphate-Buffered Saline (PBS, pH 7.4) in an ice bath (4°C). The pellets were resuspended in 3 ml PBS and subjected to ultrasonication to break the cells. The sonicated cell suspension was centrifuged (9,380 *× g* for 10 min at 4°C), and 2.8 ml of the crude enzyme extracts was added to 200 μl of AAI (0.515 mg/ml), along with NC and PBS control (the same reaction system as the crude enzyme solution). Because the suitable temperature for plant growth is 25°C and the optimal temperature for general enzyme activity is 37°C, we selected these two temperatures to compare and evaluate the AAI degradation activities of ODMs. After incubating some samples at 37°C and others at 25°C for 48 h, an equal volume of methanol was added to each sample to terminate the reaction. The samples were filtered through a 0.22 μm organic membrane and the active substances were determined using ultra-high-performance liquid chromatography (UPLC; Yin et al., 2020).

The various niches of *A. heterotropoides* were homogenized in a mortar and pestle using liquid nitrogen. 0.4 g of the homogenate was mixed with 5 ml of 70% methanol (chromatography grade) to form a solution that was then vortexed for 30 s, left standing for 1 h, and weighed. After ultrasonication for 1 h, the solution was weighed again and the weight loss was compensated by 70% methanol. The supernatant of the solution was filtered through a 0.22 μm organic membrane into a vial and stored airtight at 4°C (Yin et al., 2020).

To determine the contents of AAI, we performed UPLC on a Waters e2695/ Acquity H-class system with a Waters AcQUITY UPLC^®^ BEH C18 column (100 × 2.1 mm, 1.7 μm; Waters, Milford, MA, United States). The chromatographic settings for determining the AAI (Cas:313–67-7, purity ≥98%, Shanghai Yuanye Bio-Technology Co., Ltd., Shanghai, China) contents were as follows: mobile phase, acetonitrile: 0.1% aqueous formic acid (35, 65); column temperature, 27°C; flow rate, 0.4 ml/min; detection wavelength, 254 nm; and injection volume, 2 μl. The retention time was determined using standard samples, and the AAI content of each tissue sample was determined using a linear regression equation. Each sample was analyzed in triplicate to ensure data repeatability.

High-resolution mass spectrometric conditions: Electric spray ion source (ESI); Scanning mode: Positive ion mode; Dry gas: nitrogen; Capillary voltage:3.2 kV; Nitrogen flow rate:800 l/h; Ion source temperature:150°C; Dry gas temperature: 550°C; Impact voltage: 30 V. Model of mass spectrometer: Q-Exactive Plus (Thermo Scientific, United States; Yuan et al., 2007).

### Statistical analysis

One-way analysis of variance (ANOVA) was conducted to detect significant differences among treatments. Separation of means was tested using the least means square difference with a significance level of *p* < 0.05. All the data were presented as means of three replicates with a standard error unless stated otherwise.

## Results

### Identification and verification of AAI degradation by endophytic fungus

**Figure 3:**
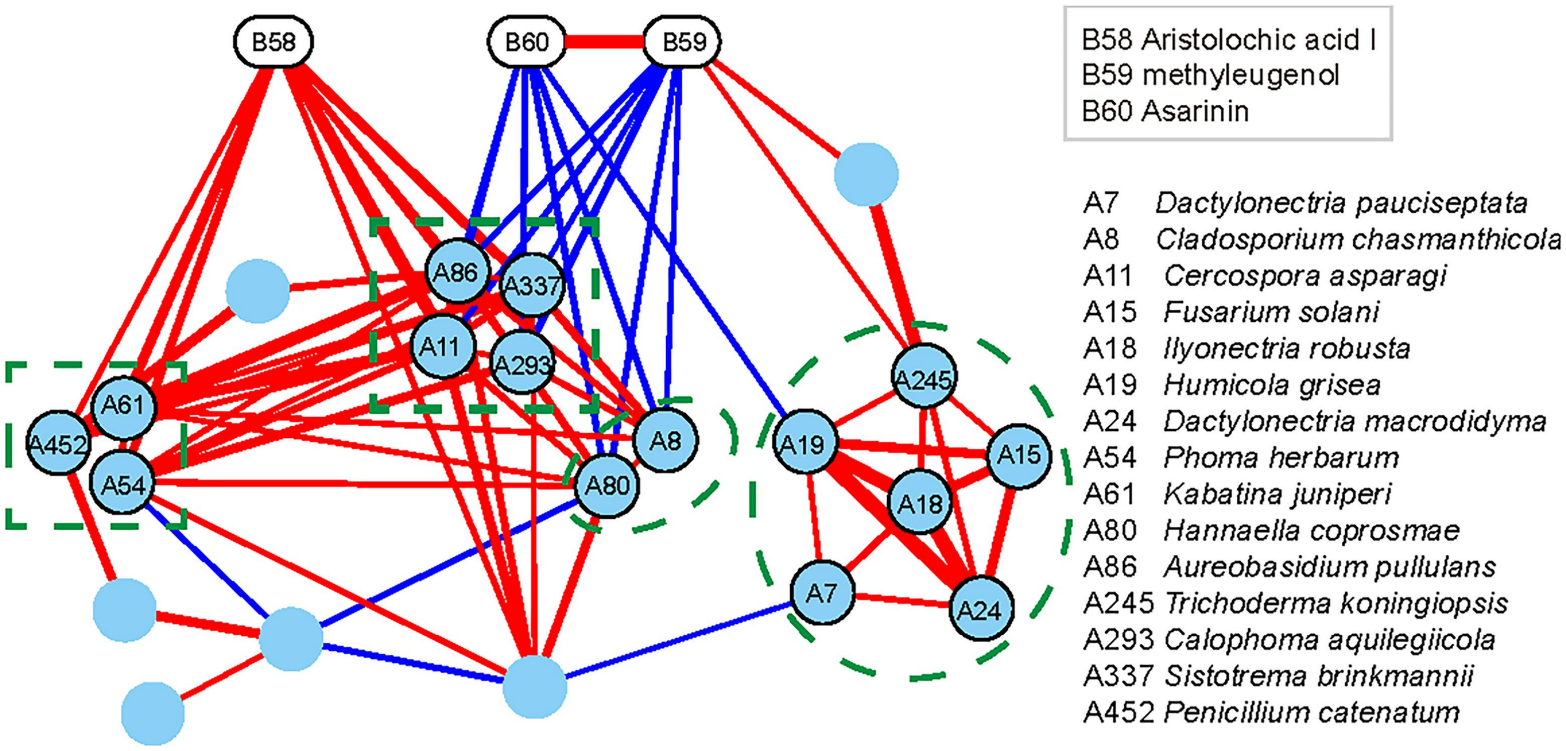
Interaction network of differential functional groups in different niches of *A. heterotropoides*. Pearson correlation between the contents of the three active ingredients and the relative contents of the endophytic fungi in different niches of *A. heterotropoides*. Endophytic fungi with significant correlation (*p* < 0.05) were used as nodes to construct the interaction network and visualization in Cytoscape. Red lines indicate a positive correlation, whereas blue lines indicate a negative correlation. Line width is proportional to the values of Pearson correlation coefficient. Raw data is provided in the EXCEL 1-ITS.xlsx.

The changes in the content of AAI in all the niches were found similar to those of some endophytic fungi of *A. heterotropoides* plants. Thus, endophytic fungi of *A. heterotropoides* may affect the synthesis and decomposition of active ingredients, including AAI. Further, we determined the diversity of endophytic fungi and contents of active ingredients in *A. heterotropoides* from different ecological niches. By analyzing the differences among active ingredient contents and endophytic fungus distributions, we identified differential fungal species that exhibited significant correlations with active ingredients (Figure 3).

**Figure 4:**
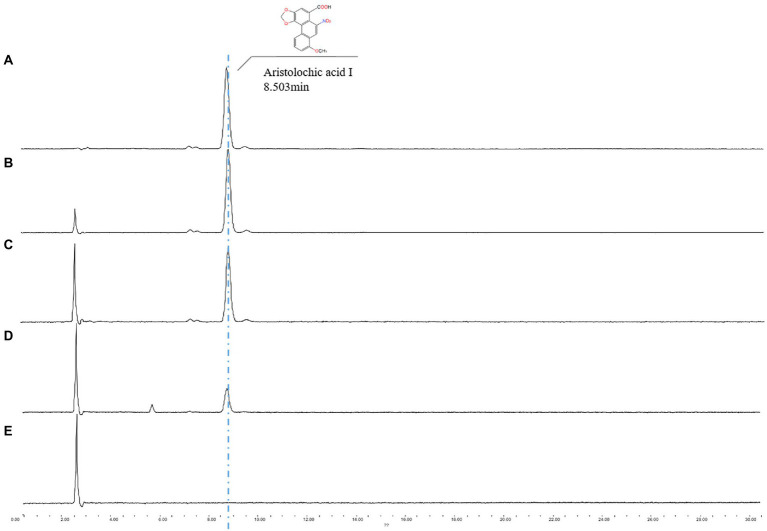
UPLC spectrum analysis of A.h-Fs-1 degradation of AAI. **(A)** Minimal medium containing AAI (0.515 mg/ml). **(B)** Minimal medium containing AAI for a degradation time of 14 days. **(C)** Minimal medium containing AAI and A.h-Fs-1 for a degradation time of 5 days. **(D)** Minimal medium containing AAI and A.h-Fs-1 for a degradation time of 14 days. **(E)** Minimal medium.

**Figure 5:**
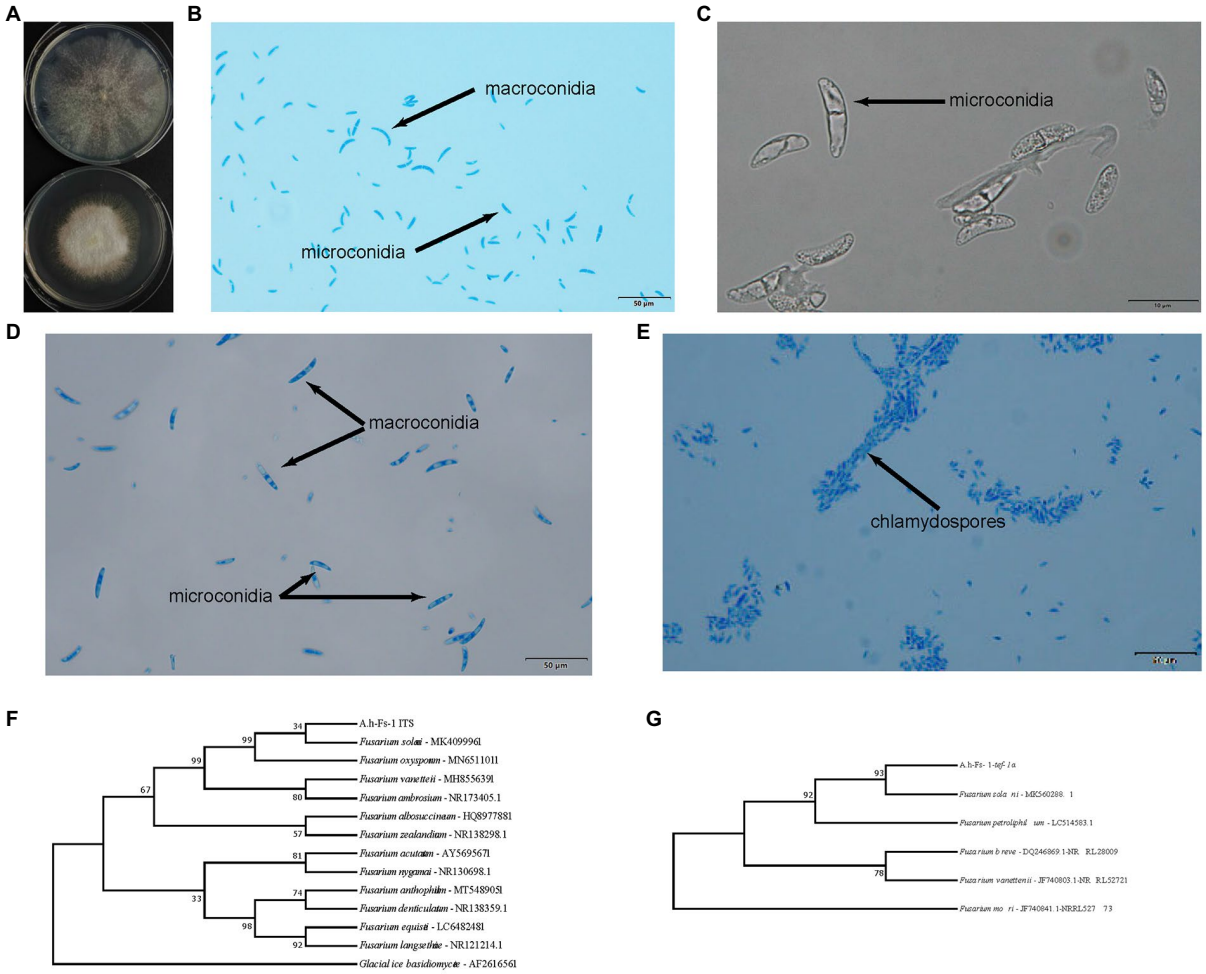
Identification of A.h-Fs-1 (*Neocosmospora solani*). **(A)** A.h-Fs-1 cultured on plate medium (top: A.h-Fs-1 on the AAI-coated inorganic salt medium plate; bottom: A.h-Fs-1 on PDA medium plate). **(B)** The microconidia and macroconidia of A.h-Fs-1 cultured on the AAI-coated inorganic salt medium plate. **(C)** The microconidia of A.h-Fs-1 cultured on the AAI-coated inorganic salt medium plate. **(D)** The microconidia and macroconidia of A.h-Fs-1 cultured on PDA medium plate. **(E)** Chlamydospores and hyphae of A.h-Fs-1. **(F)** ITS phylogenetic tree of A.h-Fs-1; **(G)** The *tef-1α* phylogenetic tree of A.h-Fs-1.

One endophytic fungus, namely A.h-Fs-1, was identified upon screening for a strain that could degrade AAI. Next, we tested the AAI degradation function of A.h-Fs-1 using UPLC. The 14-day culture of AAI degradation showed a high and stable degradation efficiency, at 28.85% on day 5 and 64.42% on day 14 (Figure 4). We performed morphological, microscopic, and internal transcribed spacer molecular analyses to evaluate A.h-Fs-1. On the potato dextrose agar medium plates, the colony of degrading endophytic fungus showed flat and villus shape with smooth edges, and it grew rapidly (Figure 5A). The hyphae were hyaline, septate, and branched under a microscope. The microconidia had 1–2 compartments and appeared as spindle or ovoid (Figures 5B–D); the macroconidia had 3–5 compartments and appeared as prismatic or crescent with a little blunt end (Figures 5B,D). Chlamydospores grew at the hyphal apex and appeared round (Figure 5E). On AAI-coated inorganic salt solid medium plates, the colony of this degrading fungus grew slowly with loose aerial mycelia (Figure 5A) and a bit of sharp macroconidia ends (Figure 5B), and the microconidia and chlamydospore morphologies were the same as those on the potato dextrose agar medium. Comparison of the sequencing results of this AAI-degrading fungus using BLAST and ITS phylogenetic tree analysis indicated 100% similarity to *N. solani* (Figure 5F). The *tef-1α* phylogenetic tree showed a strong relationship between A.h-Fs-1 and *N. solani* (Figure 5G). Therefore, both the 100% similarity and the strong relationship helped to identify the endophytic fungus as *N. solani*.

### ODM cloning and homology sequence analysis

**Figure 6:**
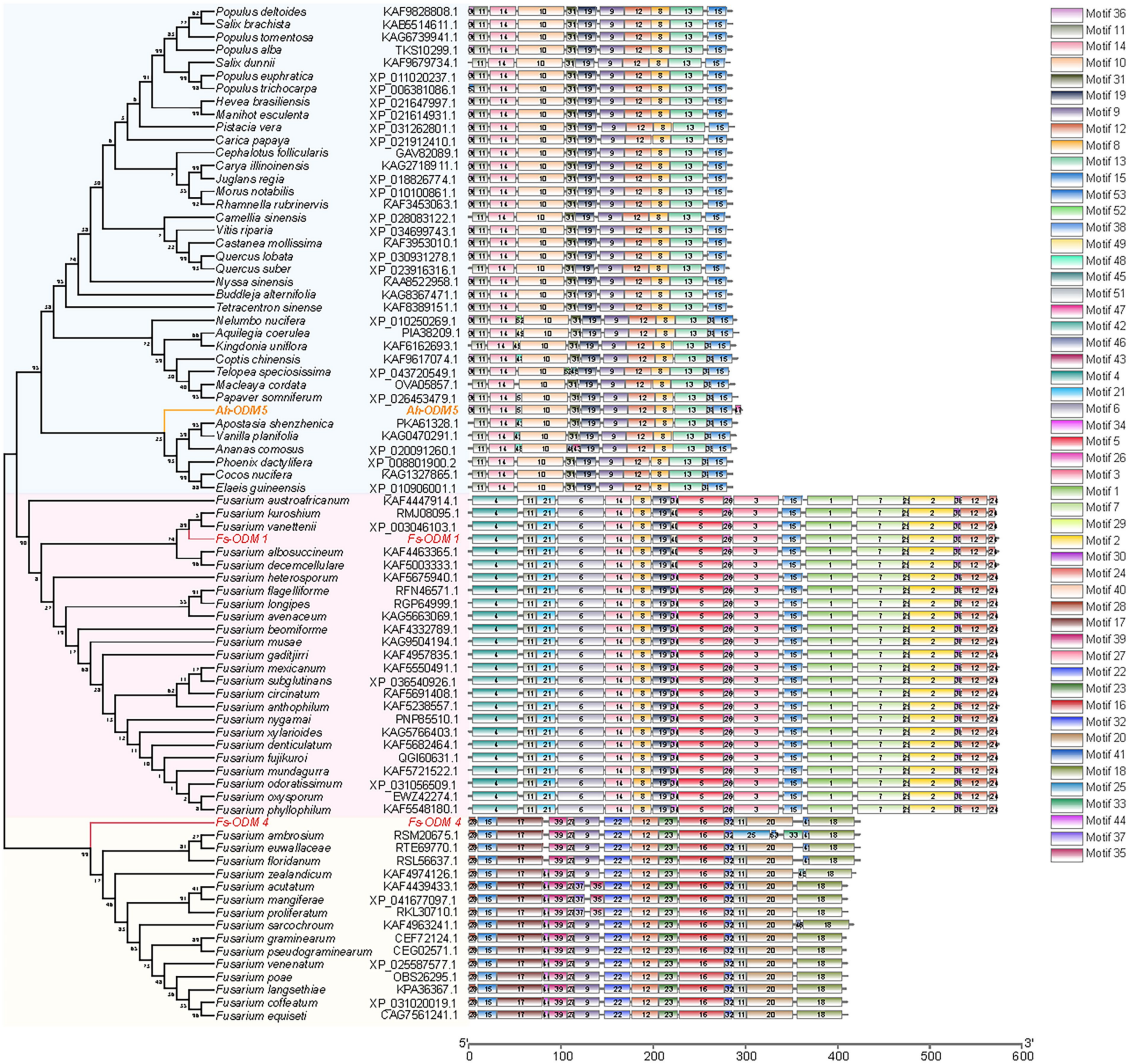
Comparison of sequence motifs of ODMs and developmental tree analysis of *ODMs*. The amino acid sequences of ODMs of 37 species of plants are shown in light blue block, and the amino acid sequences of ODMs of 39 species of *Fusarium* strains are shown in pink (*Fs*-ODM-1) and light yellow (*Fs*-ODM-4) blocks. Raw data are provided in EXCEL 2.xlsx.

The candidate protein involved in AAI degradation was found to be demethylase by analyzing the molecular structure and carcinogenic properties of AAI. We performed bioinformatic and structural analysis (Supplementary Material 2) of the *A. heterotropoides* transcriptome and *N. solani* genome, and screened nine *ODMs* (five *ODMs* from the plant and four *ODMs* from endophytic fungi). However, after numerous trials of adjusting primers and cloning, only three genes were successfully cloned, including one from plant (*A.h-ODM-5*) and two from endophytic fungi (*Fs-ODM-1* and *Fs-ODM-4*). Therefore, these three genes were further analyzed in subsequent experiments. *A.h-ODM-5* (*A. heterotropoides*), *Fs-ODM-1*(*N. solani*), and *Fs-ODM-4*(*N. solani*) were identified for cloning and *in vitro* verification. Bioinformatic analysis revealed that the open reading frames of *A.h-ODM-5*, *Fs-ODM-4*, and *Fs-ODM-1* were 891, 1,275, and 1725 bp in length (Supplementary Figure S3A), encoding 296, 424, and 574 amino acids, respectively. The nucleotide and amino acid sequences of the *ODMs* were compared using BLASTX (GenBank), and the amino acid sequences of other strains and plants were analyzed using DNAMAN software (Supplementary Figures S3B,C). We selected the amino acid sequences of *ODMs* from 37 species of plants and 39 species of *Fusarium* strains recorded in GenBank, and constructed a phylogenetic tree using the maximum likelihood method in MEGA7.0 (Figure 6).

### Evaluation of AAI degradation by ODM enzyme

**Figure 7:**
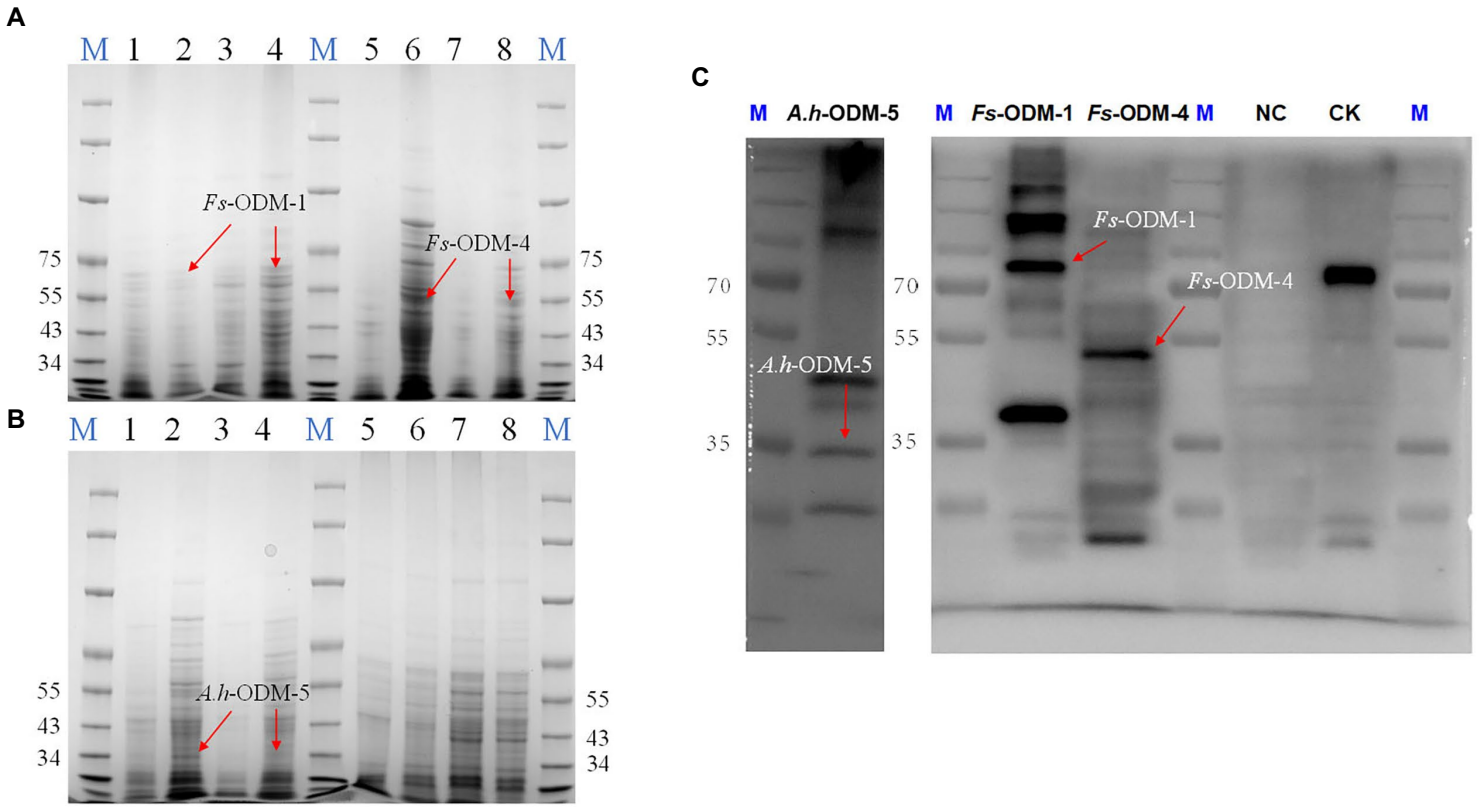
SDS-PAGE and WB detection of ODMs recombinant protein. **(A)** SDS-PAGE detection of *Fs*-ODM-1, *Fs*-ODM-1Y (induced), *Fs*-ODM-4, and *Fs*-ODM-4Y (induced) recombinant protein (columns 1–8 correspond to *Fs*-ODM-1-37, *Fs*-ODM-1-37Y, *Fs*-ODM-1-16, *Fs*-ODM-1-16Y, *Fs*-ODM-4-16, *Fs*-ODM-4-16Y, *Fs*-ODM-4-37, and *Fs*-ODM-4-37Y, respectively); **(B)** SDS-PAGE detection of *A.h*-ODM-5, *A.h*-ODM-5Y (induced), NC, and NCY (induced) recombinant protein(columns 1–8 correspond to *A.h*-ODM-5-16, *A.h*-ODM-5-16Y, *A.h*-ODM-5-37, *A.h*-ODM-5-37Y, NC-16, NC-16Y, NC-37, and NC-37Y, respectively); **(C)** Western blot (WB) detection of *A.h*-ODM-5-16Y(induced), *Fs*-ODM-1-16Y(induced), *Fs*-ODM-4-16Y(induced), and NC-16Y (induced) recombinant protein. Notes: “−16” means culture at 16°C and “−37” means culture at 37°C. CK was used recombinant 16 tag protein produced by Lianmai Biology. It is a recombinant protein with a size of 68.5 kd and is expressed in *E. coli*. It is often used as a positive control for WB detection of any of the 16 Tags (include His-tag).

**Figure 8:**
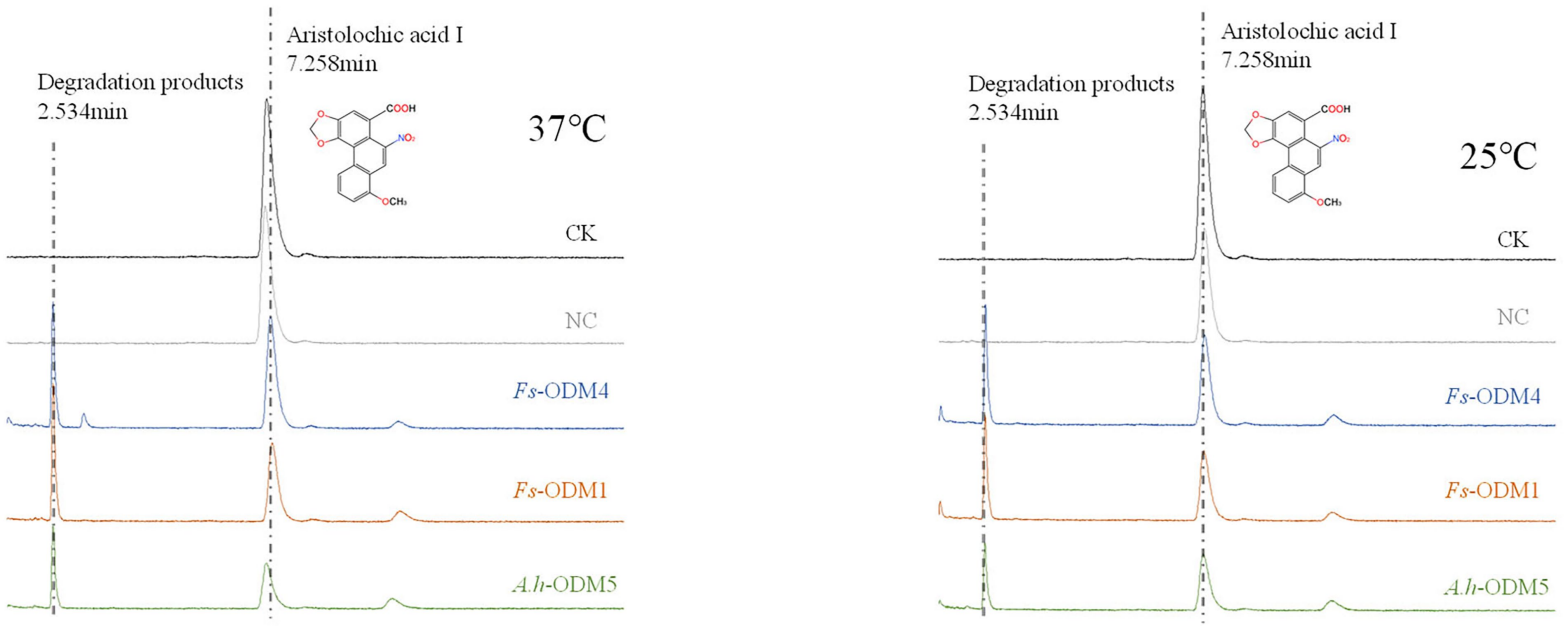
UPLC profile of AAI degraded by ODMs enzyme at 37°C (left) and 25°C (right). The degradation efficiency of three ODMs is shown in Supplementary Table S3.

Under different induction conditions of 16°C and 37°C, we used SDS-PAGE to evaluate the expression levels of three *ODMs* in *E. coli* (Figures 7A,B). In *E. coli* cultures expressing *ODM-TransB* variants, recombinant protein was detected in the supernatants. The expression levels of three *ODMs* at 16°C in *E. coli* were higher than that at 37°C, so we took 16°C as the induction condition of bacterial solution. At the same time, we used Western blot (WB) to detect the size and expression of the three enzymes (Figure 7C). The recombinant protein sizes were determined as 68.88 kDa for *Fs*-ODM-1, 50.88 kDa for *Fs*-ODM-4, and 35.52 kDa for *A.h*-ODM-5. Recombinant protein extracted from *E. coli* was used for *in vitro* enzyme assays to simulate the bio-transformation of AAI at different temperatures. The UPLC (Figure 8) results showed that the crude enzyme extract could significantly reduce the content of AAI, and there was an independent peak different from the control and blank at 2.534 min. The reaction cultured at 25°C and 37°C showed different degrees of degradation. At 25°C, ODMs showed the highest degradation efficiency, and *Fs*-ODM-4 showed the lowest efficiency at both temperatures (Figure 8; Supplementary Table S3). The temperature decrease from 37°C to 25°C promoted the efficiency of AAI degradation by *Fs*-ODM-1 and *Fs*-ODM-4, with the latter corresponding to a significant efficiency leap from 29.60 to 48.79%. The results indicated that the activity of degradation by *Fs*-ODM-1 and *Fs*-ODM-4 is temperature-sensitive and high temperature would restrain degradation. According to the comprehensive analysis of UPLC degradation results and gene annotation, we speculate that the degradation product may be 8-hydroxyaristolochic acid I. Through mass spectrometry detection and analysis, we can see that the substrate AAI parent ion [M + NH_4_] ^+^ has good stability after entering the secondary mass spectrometry, but the response value is low. LC–MS showed that the EIC response values of AAI in CK, NC, *Fs*-ODM-1, *Fs*-ODM-4, and *A.h*-ODM-5 were 1.21e8, 1.09e8, 1.65e7, 1.60e7, and 2.26e7, respectively (Supplementary Figure S6).

## Discussion

AAI, naturally present in *A. heterotropoides* that is broadly used for herbal medicines, can cause nephrotoxicity after being absorbed by the human body. Therefore, it is imperative to develop effective methods to degrade and metabolize AAI. Through bioinformatic analysis and sole carbon source experiments, we identified an AAI-degrading strain, namely A.h-Fs-1 (*N. solani*). As a fungus commonly available in soil, animals, and plants (Schroers et al., 2016), *N. solani* is both pathogenic and beneficial to the host (Karpouzas et al., 2011; Chehri et al., 2015). For example, *N. solani* can produce a variety of toxins after infestation, leading to plant root rot and reduced plant production (Postma and Scholte, 1981; Tamura et al., 2015; Bryła et al., 2020; Wang et al., 2020; Phasha et al., 2021). Additionally, the endophytic strains of *N. solani* can produce beneficial compounds, such as emodin and rhein (Yu et al., 2015). Furthermore, *N. solani* isolated by Meng et al. (2016) from the ocean can produce various secondary metabolites, such as 3-hydroxy-3-methyl glutaryl coenzyme A reductase inhibitors.

We explored the mechanism of AAI degradation by *N. solani*, and observed that the strain A.h-Fs-1 degraded 65% of the supplied AAI substrate in 14 days. Based on the structural characteristics of AAI and the availability of the genome of *N. solani*, demethylase was identified as the most likely candidate for AAI degradation. *In vitro* assays demonstrated that all three demethylases, one from *A. heterotropoides* and two from *N. solani*, can degrade AAI. Moreover, none of the three demethylases required coenzymes. The degradation product was *O*-demethylate of AAI and its analogs. This is consistent with the results obtained by Guo (2006), who used *Cunninghamella blakesleeana* AS3.910 to transform AAI. The observed degradation efficiency and time were similar to those reported by Guo (2006), who used *C. brevisiae* to biotransform AAI and AAII and performed a specific *O*-demethylation structural modification of AAI. However, the strain in our experiment isolated from the rhizome of *A. heterotropoides* is an endophytic fungus that is more conducive to back-staining and colonization, whereas *C. blakesleeana* is exophytic in *A. heterotropoides*. After it enters the human body, AAI is reduced to N-hydroxyaristolactam I, which is the activation pathway of carcinogenic effects (Pfau et al., 1990; Chang et al., 2017; Anger et al., 2020). In the detoxification pathway, the redox metabolites of AAI are excreted in the urine and feces (Shibutani et al., 2010). The oxidative demethylation product of AAI is 8-Hydroxy AAI, which may be among the metabolites obtained in some previous studies (Levová et al., 2011; Stiborová et al., 2015). The LC–MS showed that although AAI contents were reduced by an order of magnitude according to the degradation product peak, the target values of the secondary ions ([M-H_2_O-CO_2_ + NH_4_] ^+^, [M + NH_4_] ^+^, [M-H_2_O-CO_2_ + H] ^+^, and [M + H] ^+^) were not found. The reason may be that the response value of AAI in mass spectrometry is relatively low, and the response value of its degradation product peak would be too low to display. The specific reason needs to be identified in further study.

In plants, demethylases mostly regulate the methylation of DNA and histones (Qiu et al., 2019). Overexpression of plant demethylases may lead to undesirable effects. Studies of demethylase have mainly focused on the effect of methylation modification on epigenetics and some modifications of cytoplasmic proteins through methylation (Li, 2014; Mao, 2014; Ma et al., 2020). However, the heterologous overexpression of *N. solani* demethylases in plants may offer a solution to detoxification. Because *ODMs* from different sources are quite different, we compared with *A.h*-ODM-5 from *A. heterotropoides* to determine the reaction efficiency of *Fs*-ODM-4 and *Fs*-ODM-1. If overexpressed, *A.h*-ODM-5 may affect the plant appearance. Considering the controversy of transgene, it is considered that the of endophytic fungi is more practical. Demethylases from *N. solani* are closely related to plant demethylases and they can degrade AAI *via* metabolization, but they cannot enter the plant cell nucleus to participate in epigenetic regulation. *A.h*-ODM-5 is an ODM that maintains normal life activities in *A. heterotropoides*. *A.h*-ODM-5, *Fs*-ODM-1, and *Fs*-ODM-4 are no signal peptide. This is a non-secretory protein and usually not secreted outside the cell. However, studies have shown that non-secretory proteins can be secreted outside the cell in an abnormal mode, which usually occurs in fungi, especially endophytic fungi (Cohen et al., 2020; Nguyen and Debnath, 2020; Zhang et al., 2020; Niu et al., 2021). Therefore, we speculated that *Fs*-ODM-1 and *Fs*-ODM-4 can be transported outside fungal cells or inside plant cells through other secretory pathways to degrade AAI. We used *A.h*-ODM-5 as a reference for the *in vitro* activities of *Fs*-ODM-1 and *Fs*-ODM-4. *A.h*-ODM-5 is similar to the internal reference of *A. heterotropoides*. The results showed that the degradation efficiency of *Fs*-ODM-1 and *Fs*-ODM-4 for AAI was close to that of *A.h*-ODM-5. Therefore, the strain would offer insights into follow-up re-staining and in-depth study. Although these genes are quite different in homology, they play the same role in the structural modification of methyl-containing secondary metabolites, such as AAI. Our results also demonstrate that methylation plays a role in protein–protein interactions and affects protein stability and enzyme activity. These results indicate that demethylases are multifunctional proteins that are involved in epigenetics and the structural modification of secondary metabolites.

## Conclusion

In this study, we identified an endophytic fungus that is able to decompose the toxic compound AAI of *Asarum* spp., and screened for candidate genes involved in AAI degradation. The mechanism of AAI degradation by A.h-Fs-1 was uncovered and elaborated. The analysis of *N. solani* genome and *A. heterotropoides* transcriptome database indicated demethylases as candidate proteins involved in AAI degradation. We cloned *ODMs* and used them for *in vitro* assays, which validated the capability of *ODMs* to degrade AAI. Our results show that non-histone ODMs play an important role in detoxifying herbal medicine. Further studies on the specific activity and process of AAI degradation by A.h-Fs-1 would facilitate the development of detoxification approach, and provide promising insights into regulation of the content of toxic ingredients in the source materials for herbal medicine.

## Data availability statement

The original contributions presented in the study are included in the article/Supplementary material; further inquiries can be directed to the corresponding authors.

## Author contributions

XW and CL conceived and designed the study. XW and QS performed all analyses. XW analyzed the data and prepared the manuscript. XW, CL, GR, and DJ contributed to the writing of the manuscript. All authors contributed to the article and approved the submitted version.

## Funding

This research was funded by the National Natural Science Foundation of China (nos. 81773838 and 82104327) and the Fundamental Research Funds for the Central Universities (Beijing University of Chinese Medicine, no. 2020-JYB-ZDGG-037).

## Conflict of interest

The authors declare that the research was conducted in the absence of any commercial or financial relationships that could be construed as a potential conflict of interest.

## Publisher’s note

All claims expressed in this article are solely those of the authors and do not necessarily represent those of their affiliated organizations, or those of the publisher, the editors and the reviewers. Any product that may be evaluated in this article, or claim that may be made by its manufacturer, is not guaranteed or endorsed by the publisher.

## Footnotes

^1^ http://www.ncbi.nlm.nih.gov/

^2^ https://www.ncbi.nlm.nih.gov/orffinder/

^3^ https://web.expasy.org/protparam/

^4^ http://www.cbs.dtu.dk/services/TMHMM/

^5^ http://www.cbs.dtu.dk/services/SignalP/

^6^ https://www.ncbi.nlm.nih.gov/cdd

^7^https://npsa-prabi.ibcp.fr/cgi-bin/npsa_automat.pl?page=npsa_sopma.html

^8^ http://zhanggroup.org//I-TASSER/

^9^ http://swissmodel.expasy.org/

^10^ http://meme-suite.org/tools/meme
