# Supplementary material for: Microbial degradation of aristolochic acid I by endophytic fungus A.h-Fs-1 of Asarum heterotropoides
Source: Front Microbiol. 2022 Jul 22;13:917117. doi: 10.3389/fmicb.2022.917117 (PMC9355669; doi:10.3389/fmicb.2022.917117)
Supplement: Supplementary file 1 [file Data_Sheet_1.zip › Supplementary Material/Supplementary Figures 1 - 6.docx]

Figure S1


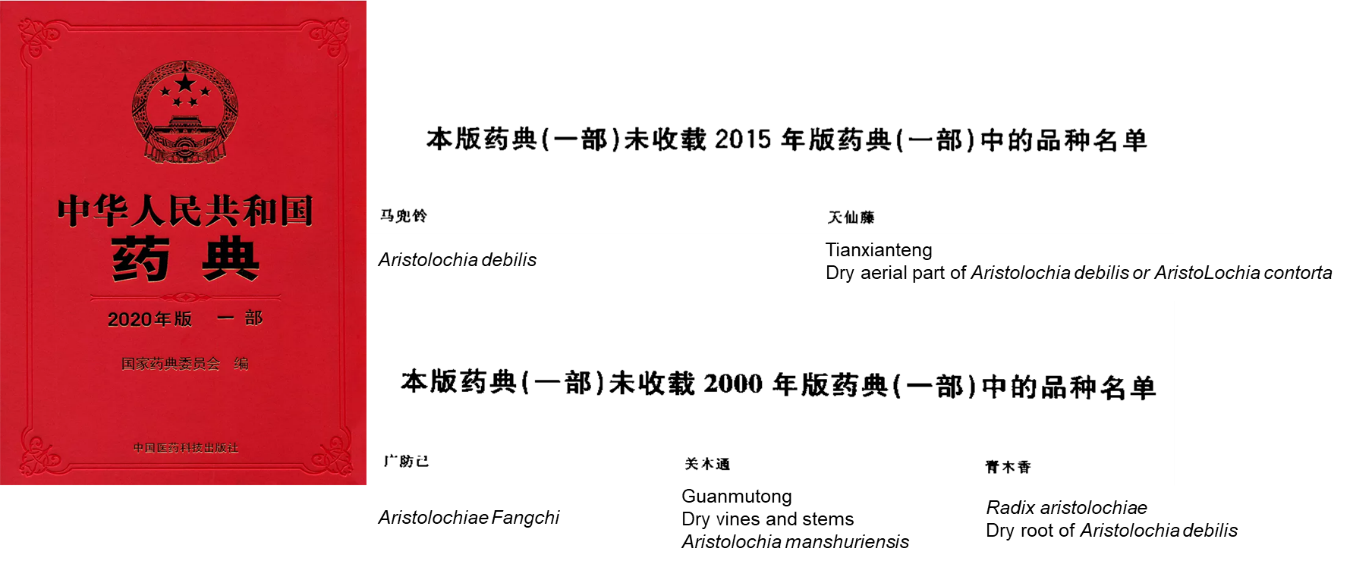


**Figure S1.** Changes in regulations in using *Aristolochiaceae* plants for medicines in *Chinese Pharmacopoeia*, 2020.

Figure S2

**Figure S2.** Chemical structure (upper) of aristolochic acid I and the photo (lower) of the plant *Asarum heterotropoides*.

Figure S3


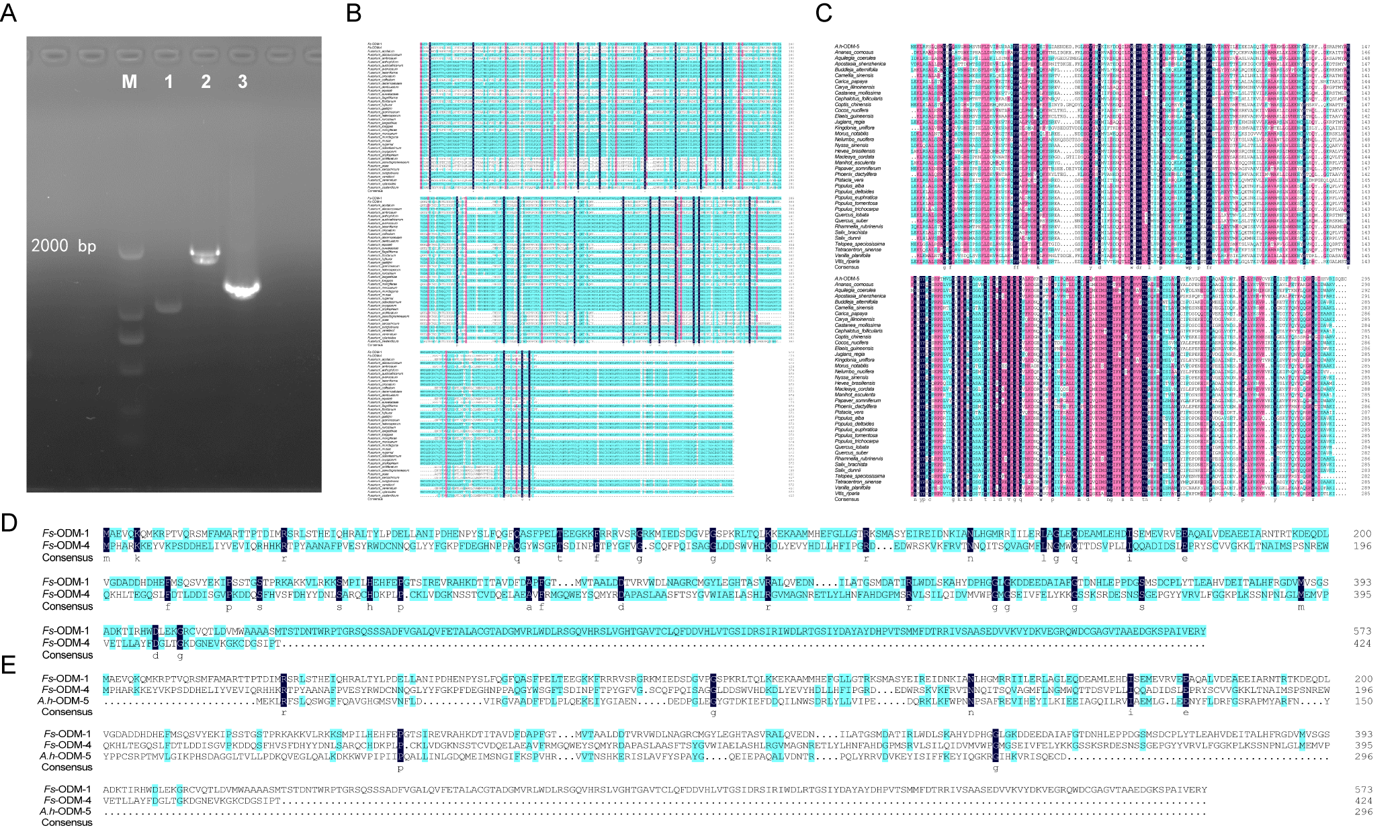


**Figure S3.** Cloning and sequence alignment of ODMs.

(A) 1, 2, and 3 correspond to *A.h*-*ODM-5*(891bp), *Fs*-*ODM-1*(1725bp), and *Fs*-*ODM-4*(1275 bp), respectively. (B) Amino acid sequence alignment of *Fusarium*. (C) Amino acid sequence alignment of plant. (D) Amino acid sequence alignment of *Fs*-ODM-1 and *Fs*-ODM-4. (E) Amino acid sequence alignment of *A.h*-ODM-5, *Fs*-ODM-1 and *Fs*-ODM-4, respectively.

Figure S4

**Figure S4.** ODM domain analysis, transmembrane domain analysis and signal peptide analysis.

(A) ODM domain analysis (1, 2, and 3 correspond to *Fs*-ODM1, *Fs*-ODM4, and *A.h*-ODM5, respectively). (B) ODM transmembrane domain analysis (1, 2, and 3 correspond to *Fs*-ODM1, *Fs*-ODM4, and *A.h*-ODM5, respectively). (C) ODM signal peptide analysis (1, 2, and 3 correspond to *Fs*-ODM1, *Fs*-ODM4, and *A.h*-ODM5, respectively).

Figure S5

**Figure S5.** Prediction of secondary and tertiary structures of ODMs.

(A) Secondary structure of ODMs (1, 2, and 3 correspond to *Fs*-ODM-1, *Fs*-ODM-4, and *A.h*-ODM-5, respectively). (B) Tertiary structure of ODMs (1, 2, and 3 correspond to *Fs*-ODM-1, *Fs*-ODM-4, and *A.h*-ODM-5, respectively).

Figure S6


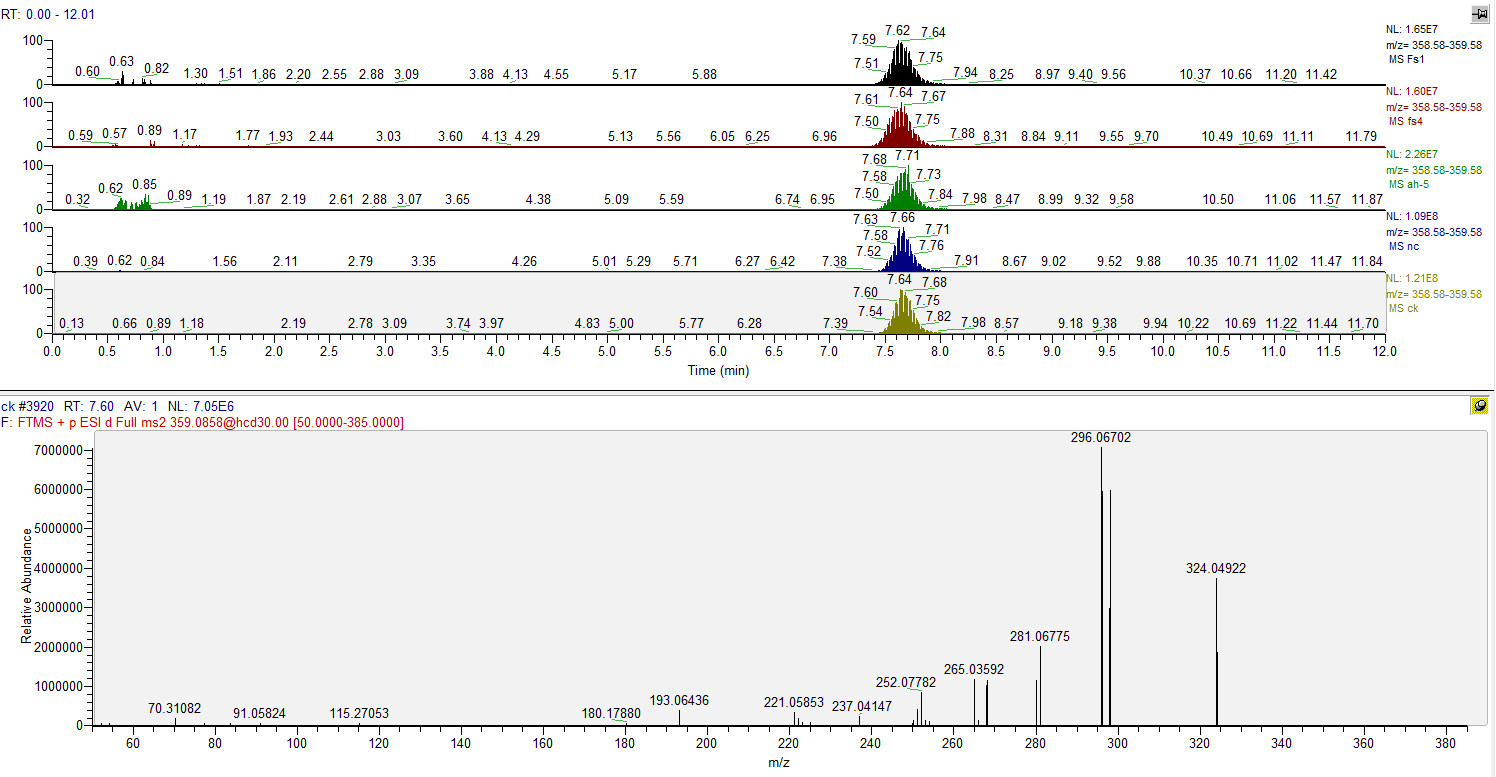


**Figure S6.** High resolution mass spectrogram analysis.
