# Supplementary material for: Microbial degradation of aristolochic acid I by endophytic fungus A.h-Fs-1 of Asarum heterotropoides
Source: Front Microbiol. 2022 Jul 22;13:917117. doi: 10.3389/fmicb.2022.917117 (PMC9355669; doi:10.3389/fmicb.2022.917117)
Supplement: Supplementary file 1 [file Data_Sheet_1.zip › Supplementary Material/Supplementary Material 1.docx]

Supplementary Material 1

**Methods**

**Extraction and sequencing**

Total genomic DNA was extracted using the CTAB method (Allen et al., 2006). DNA concentration and purity were determined on 1% agarose gels. The DNA was diluted to 1 ng/μL with sterile water. The ITS genes in distinct regions were amplified with specific barcoded primers (ITS1: CTTGGTCATTTAGAGGAAGTAA; ITS4: GCTGCGTTCTTCATCGATGC). All polymerase chain reaction (PCR) reactions were performed using 15 μL Phusion^®^ High-Fidelity PCR Master Mix (New England Biolabs, Ipswich, MA, USA), 2 μM of each forward and reverse primer (Sangon Biotech, Shanghai, China), and 10 ng template DNA. Thermal cycling of ITS consisted of an initial denaturation at 98 °C for 1 min followed by 30 cycles of denaturation at 98 °C for 10 s, annealing at 50 °C for 30 s, elongation at 72 °C for 30 s, and a final extension period of 5 min at 72 °C. Equal volumes of 1× loading buffer containing SYBR green and the PCR products were subjected to electrophoresis on 2% agarose gel. The PCR products were mixed at equal densities. The PCR products were purified with a gel extraction kit (Qiagen, Hilden, Germany).

Sequencing libraries were generated with a TruSeq^®^ DNA PCR-free sample preparation kit (Illumina, San Diego, CA, USA) following the manufacturer instructions and index codes were added. The library quality was assessed in a Qubit@ 2.0 fluorometer (Thermo Fisher Scientific, Waltham, MA, USA) and an Agilent Bioanalyzer 2100 system (Agilent Technologies, Santa Clara, CA, USA). The library was sequenced on an Illumina NovaSeq platform (Illumina, San Diego, CA, USA) and 250-bp paired-end reads were generated.

**Quality control**

Paired-end reads were assigned to samples based on their unique barcodes, truncated by cutting off the barcodes and the primer sequences, and merged with FLASH v. 1.2.7 (http://ccb.jhu.edu/software/FLASH/) (Magoč and Salzberg, 2011). This programme is a rapid and accurate analytical tool that merges paired-end reads when some of them overlap the reads generated at opposite ends of the same DNA fragment. The splicing sequences are called raw tags.

Quality filtering of the raw tags was conducted under specific filtering conditions. High-quality clean tags were acquired via the QIIME v. 1.9.1 (http://qiime.org/scripts/split_libraries_fastq.html) quality control process. The tags were compared against the reference Silva database (https://www.arb-silva.de/) using the UCHIME algorithm (http://www.drive5.com/usearch/manual/uchime_algo.html), and chimaera sequences were detected and removed (Caporaso et al., 2010; Edgar et al., 2011; Haas et al., 2011; Bokulich et al., 2013).

**Operational taxonomic unit (OTU) cluster and species annotation**

Sequence analysis was performed with UPARSE v. 7.0.1001 (http://drive5.com/uparse/) (Edgar, 2013). Sequences with ≥ 97% similarity were assigned to the same OTUs. Representative OTU sequences were screened for further annotation. The Silva database (http://www.arb-silva.de/)(Quast et al., 2012) annotated taxonomic information according to the Mothur algorithm. To study the phylogenetic relationships among various OTUs and identify the dominant species in different samples (groups), a multiple sequence alignment was conducted using MUSCLE v. 3.8.31 (http://www.drive5.com/muscle/)(Edgar, 2004). The OTU abundance data were normalised with a standard sequence number corresponding to the sample with the fewest sequences. Alpha and beta diversities were analysed on the basis of normalised output data.

**Data analysis**

Data were analysed using the *agricolae* and *vegan* libraries in R v. 2.15.3 (R Core Team, Vienna, Austria). The normality of each variable was tested and converted to logarithmic form as required. If the data were normally distributed and the variances were equal, analysis of variance (ANOVA) was performed on certain variables and factors in R v. 2.15.3 (R Core Team, Vienna, Austria), SPSS v. 25.0 (IBM Corp., Armonk, NY, USA), and SigmaPlot v. 14.0 (Systat Software Inc., Chicago, IL, USA). Data were converted to logarithmic or square root forms if they failed to meet the assumption of equal variance. The converted data were analysed by parametric tests. If the converted data failed to meet the assumption analysis, they were subjected to nonparametric tests followed by pairwise comparison post-hoc tests in SigmaPlot v. 14.0 (Systat Software Inc., Chicago, IL, USA). The specific statistical methods used for each dataset are described in each figure legend with the corresponding *P* values.

Two-tailed Wilcoxon rank-sum tests were performed using the Wilcoxon test function in the *vegan* package of R v. 2.15.3 (R Core Team, Vienna, Austria). Variations in soil chemical properties and microbial community diversity indices under different treatments and sampling times were analysed by ANOVA in SPSS v. 25.0 (IBM Corp., Armonk, NY, USA) (n = 9). Differences were compared using the least significant difference (LSD) test at the 0.05 probability level. Correlation coefficients and significance were calculated in SPSS v. 25.0 (IBM Corp., Armonk, NY, USA). Significant differences between treatments in terms of fungal taxa were identified by linear discriminant analysis (LDA) and Linear discriminant analysis Effect Size (LEfSe) statistical analysis. The correlation network was visualised in Cytoscape v. 3.8.1 (Institute for Systems Biology, North Seattle, WA, USA).

**Extraction and estimation of aristolochic acid I, methyl eugenol and asarinin** (Wang, 2014; Ma, 2016).

The plant materials were pulverised with liquid nitrogen, and 0.4 g of the powdered mixture was placed in a labelled 10 mL centrifuge tube, which was vortexed with 5 mL fresh 70% methanol (chromatography grade) for 30 s, kept aside for 1 h, and then weighed. After ultrasonication for 1 h, the tube was removed and then weighed again after cooling. The weight loss was compensated for by adding 70% methanol. The supernatant was filtered through a 0.22 μm organic membrane into a vial and stored airtight at 4 °C in the refrigerator. The fresh leaves and roots were dried to a constant weight. The extraction was performed using the extraction method described in the section of extraction and sequencing. High-performance liquid chromatography (HPLC) detection was performed using a Waters e2695/ Acquity H-class system with a ZORBAX Eclipse XDB-C18 column (250 × 4.6 mm, 5 μm; Agilent Technologies, California, USA).

Chromatographic conditions for determining aristolochic acid I (Cas:313-67-7, purity ≥98%, Shanghai Yuanye Bio-Technology Co., Ltd, Shanghai, China) content were as follows: mobile phase, methanol–0.1% aqueous acetic acid (70:30); column temperature, 27 °C; flow rate, 1.0 mL/min; detection wavelength, 250 nm; and injection volume, 10 μL. The retention time was determined using standard samples, and the aristolochic acid I content of each tissue sample was determined using a linear regression equation. Each sample was analysed in triplicate.

Chromatographic conditions for determining methyl eugenol and asarinin (Cas:93-15-2 and Cas:133-04-0, purity ≥98%, Shanghai Yuanye Bio-Technology Co., Ltd, Shanghai, China) content were as follows: mobile phase, A(methanol)–B(water), 0-10min:55-60B, 10-15min: 60-65B, 15-30min: 65-75B; column temperature, 35 °C; flow rate, 1.0 mL/min; detection wavelength, 285 nm; and injection volume, 10 μL. The retention time was determined using standard samples, and the methyl eugenol and asarinin content of each tissue sample were determined using a linear regression equation. Each sample was analysed in triplicate.

**References**

Allen, G.C., Flores-Vergara, M.A., Krasnyanski, S., Kumar, S., Thompson, W.F. (2006). A modified protocol for rapid DNA isolation from plant tissues using cetyltrimethylammonium bromide. *NAT. PROTOC*. 1(5): 2320.

Bokulich, N.A., Subramanian, S., Faith, J.J., Gevers, D., Gordon, J.I., Knight, R., et al. (2013). Quality-filtering vastly improves diversity estimates from Illuminaamplicon sequencing. *NAT. METHODS*. 10(1), 57-59.

Caporaso, J.G., Kuczynski, J., Stombaugh, J., Bittinger, K., Bushman, F.D., Costello, E.K., et al. (2010). QIIME allows analysis of high-throughput community sequencing data. *NAT. METHODS*. 7(5), 335-336.

Edgar, R.C. (2004). MUSCLE: multiple sequence alignment with high accuracy and high throughput. *NUCLEIC ACIDS RES*. 32(5), 1792-1797.

Edgar, R.C., Haas, B.J., Clemente, J.C., Quince, C., Knight, R. (2011). UCHIME improves sensitivity and speed of chimera detection. *BIOINFORMATICS*. 27(16), 2194-2200.

Edgar, R.C. (2013). UPARSE: highly accurate OTU sequences from microbial amplicon reads. *NAT. METHODS*. 10(10), 996-998.

Haas, B.J., Gevers, D., Earl, A.M., Feldgarden, M., Ward, D.V., Giannoukos, G., et al. (2011). Chimeric 16S rRNA sequence formation and detection in Sanger and 454-pyrosequenced PCR amplicons. *GENOME RES*. 21(3), 494-504.

Ma, L. (2016) Effects of amino acids, PGRs and E0 gene on accumulations of secondary metabolisms in *Asarum heterotropoides* Fr. Schmidt var. *mandshuricum* (Maxim.) Kitag. [master’s thesis]. [Changchun (Jilin)]: Jilin Agricultural University

Magoč, T., Salzberg, S.L. (2011). FLASH: fast length adjustment of short reads to improve genome assemblies. *BIOINFORMATICS*. 27(21), 2957-2963.

Quast, C., Pruesse, E., Yilmaz, P., Gerken, J., Schweer, T., Yarza, P., et al. (2012). The SILVA ribosomal RNA gene database project: improved data processing and web-based tools. *NUCLEIC ACIDS RES*. 41, 590-596.

Wang, X.H. (2014) Biosynthetic related enzymes of aristolochic acid in Asarum. [master’s thesis]. [Changchun (Jilin)]: Jilin Agricultural University
