# Supplementary material for: Microbial degradation of aristolochic acid I by endophytic fungus A.h-Fs-1 of Asarum heterotropoides
Source: Front Microbiol. 2022 Jul 22;13:917117. doi: 10.3389/fmicb.2022.917117 (PMC9355669; doi:10.3389/fmicb.2022.917117)
Supplement: Supplementary file 1 [file Data_Sheet_1.zip › Supplementary Material/Supplementary Material 2.docx]

Supplementary Material 2

# Results

**Bioinformatics and structural analysis of ODMs**

The three proteins encoded by *ODM*s are composed of 296, 424, and 574 amino acids. Domain analyses revealed that *Fs*-ODM1 belongs to the superfamily of WD40 and PRK03918, *Fs*-ODM4 to the histidine phosphatase domain family, and *Ah*-ODM5 to the PLN00417 family (Figure S4A1-3). The WD40 domain is present in a variety of eukaryotic proteins and exhibits various functions, including signal transduction, mRNA precursor processing, and adaptor/regulatory modules in cytoskeleton assembly (Stirnimann et al., 2010). The PRK03918 superfamily is involved in DNA double-strand break repair. The histidine phosphatase domain is found in diverse proteomes, mainly phosphatases (Rigden, 2020). PLN00417 is an oxidoreductase, namely the 2OG-Fe(II) oxygenase family protein.

ExPASy predictive base analysis of the physical and chemical properties of the ODM proteins showed that the isoelectric point varied from 5.39 to 5.51, indicating that the encoded protein is acidic (Table S4). *Fs*-ODM4 is a stable hydrophilic protein, and *Fs*-ODM1 and *Ah*-ODM5 are unstable hydrophilic proteins. None of the three ODM proteins (*Fs*-ODM1, *Fs*-ODM4, and *Ah*-ODM5) contained transmembrane domains (Figure S4B1-3). Moreover, the probabilities of *Fs*-ODM1, *Fs*-ODM4, and *Ah*-ODM5 comprised of a signal peptide were 0.060%, 0.174%, and 5.402%, respectively (Figure S4C1-3). Therefore, it is unlikely that the proteins are secreted.

Structural analyses revealed that ODMs were composed of alpha helices and random coils (Figure S5A1-3, Table S5). Tertiary structure prediction showed that the similarity between *Fs*-ODM1 and the apoptosome-procaspase-9 CARD complex (PDB ID: 3iytA) was 11.1% (Figure S5B1-3). Moreover, a similarity of 18.6% was found between *Fs*-ODM4 and the crystal structure of human lysophosphatidic acid phosphatase type 6 complexed with L-(+)-tartrate (PDB ID: 4jobA). Finally, the similarity between *Ah*-ODM5 and the crystal structure of JOX2 in complex with 2OG, Fe, and JA (PDB ID: 6lsv.2.A) was 40.1%.

**ODMs cloning and homology sequence analysis**

The results showed that the amino acid sequence of *FS*-ODM1 had 99.13% homology to *Fusarium kuroshium* (*Fk*ODM, RMJ08095.1) and 98.96% homology to *Fusarium vanettenii* (*Fv*ODM, XP_003046103.1). Moreover, *FS*-ODM4 showed 72.35% homology to *Fusarium euwallaceae* (*Fe*ODM, RTE69770.1) and 54.96% homology to *Fusarium zealandicum* (*Fz*ODM, KAF4974126.1). The amino acid sequence of *Ah*-ODM5 was 54.33% identical with that of *Nelumbo nucifera* (*Nn*ODM, XP_010250269.1), *Hevea brasiliensis* (*Hb*ODM, XP_021647997.1), *Phoenix dactylifera* (XP_008801900.2), and *Tetracentron sinense* (*Tsi*ODM, KAF8389151.1) at whereas the homology with *Aquilegia coerulea* (*Ac*ODM, PIA38209.1) and *Telopea speciosissima* (*Tsp*ODM, XP_043720549.1) was 54%. The amino acid sequence homology between *FS*-ODM1 and *FS*-ODM4 was 5.85% (Figure S4D), and that between the two *FS*-ODMs and *Ah*-ODM5 was 9.35% (Figure S4E). *FS*-ODM1 is at the same branch point as *F. vanettenii* and *F. kuroshium*; *FS*-ODM4 shares the same branch point as *F. euwallaceae*, *Fusarium floridanum* (*Ff*ODM, RSL56637.1) and *Fusarium ambrosium* (*Fa*ODM, RSM20675.1). *Ah*-ODM5 and *P. dactylifera* (*Pd*ODM, KAF9828808.1), *Ananas comosus* (*Ac*ODM, XP_020091260.1), *Cocos nucifera* (*Cn*ODM, KAG1327865.1), *Vanilla planifolia* (*Vp*ODM, KAG0470291.1), and A. *shenzhenica* (*As*ODM, PKA61328.1) are located at the same branch point.

**References:**

Rigden, D.J. (2020). “Protein Phosphohistidine Phosphatases of the HP Superfamily,” in Histidine Phosphorylation, ed. C.E. Eyers (New York, NY: Humana Press),93-107. doi:10.1007/978-1-4939-9884-5_7

Stirnimann, C.U., Petsalaki, E., Russell, R.B., Müller, C.W. (2010). WD40 proteins propel cellular networks. *TRENDS BIOCHEM. SCI*. 35(10), 565-574.
