# Supplementary material for: Microbial degradation of aristolochic acid I by endophytic fungus A.h-Fs-1 of Asarum heterotropoides
Source: Front Microbiol. 2022 Jul 22;13:917117. doi: 10.3389/fmicb.2022.917117 (PMC9355669; doi:10.3389/fmicb.2022.917117)
Supplement: Supplementary file 1 [file Data_Sheet_1.zip › Supplementary Material/Supplementary Table 1.docx]

**Table S1.**

Primer design

| Gene |  | Primer |
| --- | --- | --- |
| ITS1-1F | ITS1-1F-F | 5'- CTTGGTCATTTAGAGGAAGTAA -3' |
|  | ITS1-1F-R | 5'- GCTGCGTTCTTCATCGATGC -3' |
| *TEF-1α* | EF1H | 5'- ATGGGTAAGGAAGACAAGAC -3' |
|  | EF2T | 5'- GGAAGTACCAGTGATCATGTT -3' |
| *FS*-ODM1 | *FS*-ODM1F | 5'- ATGGCAGAACAGGAGAACCACT -3' |
|  | *FS*-ODM1R | 5'- TTACATATAACGCTCCACGATAGCTG -3' |
| *FS*-ODM4 | *FS*-ODM4F | 5'- ATGACAAGTATGGCGTTTAC -3' |
|  | *FS*-ODM4R | 5'- TCAAGTTGGGATGGAACCAT -3' |
| *A.h*-ODM5 | *Ah*-ODM5F | 5'- ATGGGTGGCGCAACGGAC -3' |
|  | *Ah*-ODM5R | 5'- TCAATCGCACTCTTGTGAAATTCTTAC -3' |

**Table S2.**

PCR amplification system

| Gene | PCR |
| --- | --- |
| *Fs*-*ODM1* | 94℃ 94℃ 58℃ 72℃ 72℃ 4℃ |
|  | 5 min 30 sec 45 sec 120 sec 10 min ∞ |
|  | 35c |
| *Fs*-*ODM4* | 94℃ 94℃ 58℃ 72℃ 72℃ 4℃ |
|  | 5 min 30 sec 45 sec 90 sec 10 min ∞ |
|  | 35c |
| *A.h*-*ODM5* | 94℃ 94℃ 55℃ 72℃ 72℃ 4℃ |
|  | 5 min 30 sec 45 sec 90 sec 10 min ∞ |
|  | 35c |
| Super-Fidelity DNA Polymerase | 95℃ 95℃ 60℃ 72℃ 72℃ 4℃ |
|  | 3 min 15 sec 15 sec 90 sec 5min ∞ |
|  | 35c |
|  | 95℃ 95℃ 56℃ 72℃ 72℃ 4℃ |
| ITS | 3 min 30 sec 30 sec 90 sec 10min ∞ |
|  | 30c |
|  | 94℃ 95℃ 59℃ 72℃ 72℃ 4℃ |
| *TEF-1α* | 3 min 30 sec 55 sec 90 sec 10min ∞ |
|  | 30c |

**Table S3.**

Degradation efficiency of ODMs

|  | 37℃ | 25℃ |
| --- | --- | --- |
| *Fs*-ODM1 | 50.23% | 57.82% |
| *Fs*-ODM4 | 29.60% | 48.79% |
| *A.h*-ODM5 | 69.74% | 66.18% |

**Table S4.**

Physical and chemical properties of ODMs

|  | Number of amino acids | Formula | Molecular weight | Theoretical pI | Instability index | Grand average of hydropathicity (GRAVY) |
| --- | --- | --- | --- | --- | --- | --- |
| *Fs*-ODM1 | 574 | C_2779_H_4402_N_808_O_880_S_31_ | 64206.27 | 5.39 | 44.35，unstable | -0.485 |
| *Fs*-ODM4 | 424 | C_2137_H_3219_N_573_O_644_S_19_ | 47850.66 | 5.51 | 35.7，stable | -0.504 |
| *A.h*-ODM5 | 296 | C_1554_H_2404_N_406_O_438_S_9_ | 34071.18 | 5.5 | 45.28，unstable | -0.251 |

**Table S5.**

The data of secondary structure of ODMs

|  | Alpha helix (Hh) | Extended strand (Ee) | Beta turn (Tt) | Random coil (Cc) |
| --- | --- | --- | --- | --- |
| *Fs*-ODM1 | 190，33.1% | 104，18.12% | 40，6.97% | 240，41.81% |
| *Fs*-ODM4 | 141，33.25% | 56，13.21% | 17，4.01% | 210，49.53% |
| *A.h*-ODM5 | 118，39.86% | 52，17.57% | 22，7.43% | 104，35.14% |
